# Do Caregiver Interventions Improve Outcomes in Relatives With Dementia? A Meta-analysis

**DOI:** 10.1093/geroni/igab046.3683

**Published:** 2021-12-17

**Authors:** Sheung-Tak Cheng

**Affiliations:** Education University of Hong Kong, The Education University of Hong Kong, Not Applicable, Hong Kong

## Abstract

Despite plenty of reviews on the benefits of nonpharmacological interventions for dementia informal caregivers, large-scale review on the effects of these interventions on the care-recipients (CRs) is lacking. We searched PsycINFO, CINAHL with Full Text, MEDLINE, and PubMed from inception to end of 2020 and found 144 articles that reported randomized controlled trials of caregiver interventions using CR outcomes. Interventions were found to reduce neuropsychiatric symptoms and mood disturbance, enhance cognition and quality of life, and delay institutionalization and mortality, with care coordination/case management, educational intervention with psychotherapeutic components, and direct training of the care-recipient (with caregiver involvement) being the more potent interventions. However, the effects were generally small to very small. Together with existing findings on caregiver outcomes, a tripartite scaffolding model of caregiver support is proposed. Future directions in terms of developing consensual guidelines, a registry of intervention manuals, and family-centered programs are discussed.

